# Expression of a long variant of CRACR2A that belongs to the Rab GTPase protein family in endothelial cells

**DOI:** 10.1016/j.bbrc.2014.11.095

**Published:** 2015-01-02

**Authors:** Lesley A. Wilson, Lynn McKeown, Sarka Tumova, Jing Li, David J. Beech

**Affiliations:** School of Medicine, University of Leeds, Leeds LS2 9JT, UK

**Keywords:** CRAC channel, calcium-release-activated calcium channel, CRACR2A, CRAC channel regulator 2A, CRACR2A-L, CRACR2A long, HUVEC, human umbilical vein endothelial cell, Calcium channel, Store-operated calcium entry, G protein, Endothelial cell, Angiogenesis

## Abstract

CRACR2A protein is described in T cells as an EF-hand-containing modulator of calcium-release-activated calcium (CRAC) channels. Here we sought relevance to calcium entry of endothelial cells. Unexpectedly, short interfering RNA designed to deplete CRACR2A had no effect on CRAC channels in endothelial cells but reduced the abundance of a protein with about twice the mass of CRACR2A. Reference to gene sequence data indicated the potential for a variant transcript encoding a C-terminal Rab GTPase extension of CRACR2A. Full-length cloning demonstrated expression of the long variant in endothelial cells. It was designated CRACR2A-L. Sequence analysis suggested it to be a previously unrecognised member of the Rab GTPase family. It made a positive contribution to endothelial tube formation. The data suggest that endothelial cells contain a long variant of CRACR2A which is an EF-hand-containing Rab protein that lacks impact on CRAC channels.

## Highlights

• Knockdown of CRACR2A lacks effect on CRAC channels in endothelial cells.
• Knockdown of CRACR2A depletes a protein twice the mass of CRACR2A.
• A long variant of CRACR2A, CRACR2A-L, occurs in endothelial cells.
• CRACR2A-L is a previously unrecognised EF-hand-containing Rab GTPase.
• CRACR2A-L has a positive role in endothelial tube formation.

## 1 Introduction

Ca^2+^-release-activated Ca^2+^ (CRAC) channels arising from Orai1 proteins mediate store-operated Ca^2+^ entry when STIM1 proteins sense Ca^2+^-depletion of the smooth endoplasmic reticulum [1,2]. A modulator of CRAC channels in T cells is CRACR2A, a 46 kDa cytosolic protein with functional N-terminal EF-hands [3].

Orai1 and STIM1 proteins are widely expressed and Ca^2+^ entry mediated by CRAC channels is suggested to be functionally important in many cell types. In endothelial cells, a positive role has been suggested in cell migration induced by the key mediator vascular endothelial growth factor [4]. Here we sought the relevance of CRACR2A to endothelial cells and CRAC channels of these cells.

## 2 Materials and methods

### 2.1 Cell culture

Human umbilical vein endothelial cells (HUVECs) (Lonza) were maintained in endothelial growth medium (EGM-2) supplemented with 2% foetal calf serum (FCS) and growth factor bullet kit (Lonza). Experiments were performed on passage 2–8 cells. Cells were maintained at 37 °C in a 5% CO_2_ incubator. Other endothelial cells were from PromoCell and maintained similarly to HUVECs.

### 2.2 Intracellular Ca^2+^ measurement

HUVECs were seeded in non-coated 96-well plates (NUNC) and incubated for 1 h in 2 μM fura-2 AM in standard bath solution (SBS) at 37 °C in the presence of 0.01% pluronic acid (Invitrogen). SBS contained (mM): NaCl 130, KCl 5, MgCl_2_ 1.2, CaCl_2_ 1.5, d-glucose 8, HEPES 10; Osmolality adjusted to 290 mOsm with NaCl; pH titrated to 7.4 with 4 M NaOH. Ca^2+^ free solution was SBS without added Ca^2+^. Cells were washed three times with SBS before measurements were made at room temperature (21 ± 2 °C) on a 96-well fluorescence plate reader (FlexStation II^384^, Molecular Devices). Fura-2 was excited at 340 and 380 nm and emission was collected at 510 nm. Readings were made every 10 s. The change (Δ) in intracellular Ca^2+^ concentration was indicated as the ratio of fura-2 emission intensities for 340 nm and 380 nm excitation (Δ*F* ratio).

### 2.3 Short interfering (si) RNA

HUVECs were transfected at 90% confluence with 20 nmol/L of CRACR2A siRNA or Orai1 siRNA (Applied Biosystems/Ambion) using Lipofectamine 2000 in OptiMEM according to the manufacturer’s instructions (Invitrogen). 4–6 h later, transfection reagents were removed and 2 mL of EGM-2 medium added. Cells were used in experiments 72 h later. CRACR2A siRNA 1 was (5′–3′) CAACAAAUCAAAAGUGAGAtt, CRACR2A siRNA 2 was GGAGUUCACUACUGGAUUUtt, and Orai1 siRNA was GGGAAGAGGAUUUUUAUAAtt. Control siRNA was a 19-bp scrambled sequence with no significant homology to human gene sequences (Silencer Negative Control number 1, Ambion).

### 2.4 Western blotting

Transfected HUVECs were harvested in PBS and lysed in buffer containing 0.5% NP40, 10 ml Tris (pH 8), 150 mM NaCl, 0.5 mM EDTA, protease inhibitor cocktail (Fermentas) and 1 mM phenylmethanesulfonylfluoride (PMSF). Equal protein amounts were loaded on 10% gels and resolved by electrophoresis. Products were transferred onto PVDF membranes and incubated overnight with primary anti-CRACR2A antibody and anti-β-actin antibody used at 1:800 and 1:2000 dilution respectively. Anti-CRACR2A was visualised using horse radish peroxidase conjugated goat anti-rabbit secondary antibody and anti-actin was visualised with anti-mouse secondary antibody and SuperSignal Femto detection reagent (PerBio Science). ImageJ was used to quantify protein band densities.

### 2.5 RT-PCR

RNA was extracted using TRI reagent (Sigma). Reverse transcription using Reverse Transcriptase (Applied Biosystems), followed by real-time PCR using SYBR green was performed using a LightCycler (Roche). The forward primer was (5′–3′) CGTCATGTACGATCTCAC and the reverse primer GTGACCAGAGTAGGCG.

### 2.6 Cloning

Reverse transcription of HUVEC total RNA was performed using Superscript II Reverse Transcriptase (Invitrogen) and a CRAC2RA-L gene specific primer (5′ GGTATGGCTGACCTTGTAGGACCCTATCA). CRACR2A-L sequence was amplified from the enriched cDNA using GoTaq Green (Promega) and cloning primers (Forward: 5′ GATCCGCTAGCGCTACACCATGGCTGCCCCTGACGGG, reverse: 5′ GGTATGGCTGACCTTGTAGGACCCTATCA) designed to be compatible with the GFP vector using Infusion-HD enzyme (Clontech). PCR reaction conditions were: 95 °C for 2 min (initial denaturation stage) followed by 40 cycles of: 95 °C for 30 s, 53 °C for 30 s, 73 °C for 2.5 min followed by a final extension at 73 °C for 5 min. CRAC2RA-L sequence was further amplified by a second round of PCR using the high fidelity DNA polymerase Phusion (Thermoscientific). Constructs were generated using the kanamycin resistant eGFP-C1 plasmid (Clontech). Constructs were verified by sequencing (Beckman Coulter).

### 2.7 Co-culture tube formation assay

Normal Human Dermal Fibroblasts were seeded into a 96 well plate (Greiner) at a density of 2 × 10^3^ cells/well and incubated at 37 °C in a 5% CO_2_ incubator for 72 h. The cell culture medium was removed and HUVECs at a density of 5.4 × 10^4^ cells/well were seeded on top of the fibroblasts in endothelial cell growth medium containing 3 ng/mL vascular endothelial growth factor and 2% FBS. Co-cultures were incubated for 7 days then fixed with ice-cold methanol for 10 min. Nonspecific binding sites were blocked with 5% donkey serum (in PBS) for 30 min at 37 °C. HUVEC tubes were visualised by staining with a primary monoclonal mouse anti-human CD31 antibody (1:500 in 1% donkey serum for 1 h at room temperature). After 3 washes in PBS a secondary Alexa Fluor 488 antibody was added for 1 h at room temperature. Plates were imaged using an IncuCyte (Essen Bioscience) and tube formation was quantified using the IncuCyte angiogenesis v2.0 software.

### 2.8 Reagents and antibodies

Fura-2 AM and Lipofectamine 2000 were obtained from Invitrogen. Polyclonal rabbit anti-CRACR2A-L antibody (15206-1-AP) from Proteintech group was used at 1:800 dilution. β-Actin (C4) mouse monoclonal IgG1 antibody (sc-47778) and bovine anti-mouse IgG-HRP (sc-2371) antibody were from Santa Cruz Biotechnology and used at 1:2000 and 1:10,000 respectively. Goat anti-rabbit HRP-conjugated antibody from Sigma was used at 1:10,000. Peroxidase-conjugated AffiniPure donkey anti-rabbit IgG (H+L) (705-035-003) from Jackson ImmunoResearch Labs Inc. was used at 1:10,000. Thapsigargin was from Sigma.

### 2.9 Data analysis

Data were analysed and figures prepared using Origin 7.5 software (OriginLab Corporation). For FlexStation experiments data are presented as *n*/*N*, which indicates the number of independent experiments (*n*) and the number of individual wells (replicates) in the 96-well plate. For Western blot data n is the number of independent experiments. Paired data sets were compared using two-tailed Student’s *t*-tests and expressed as mean ± standard error of the mean (s.e.m.). *P* < 0.05 indicated statistically significant difference (*).

## 3 Results

**Fig. 1:**
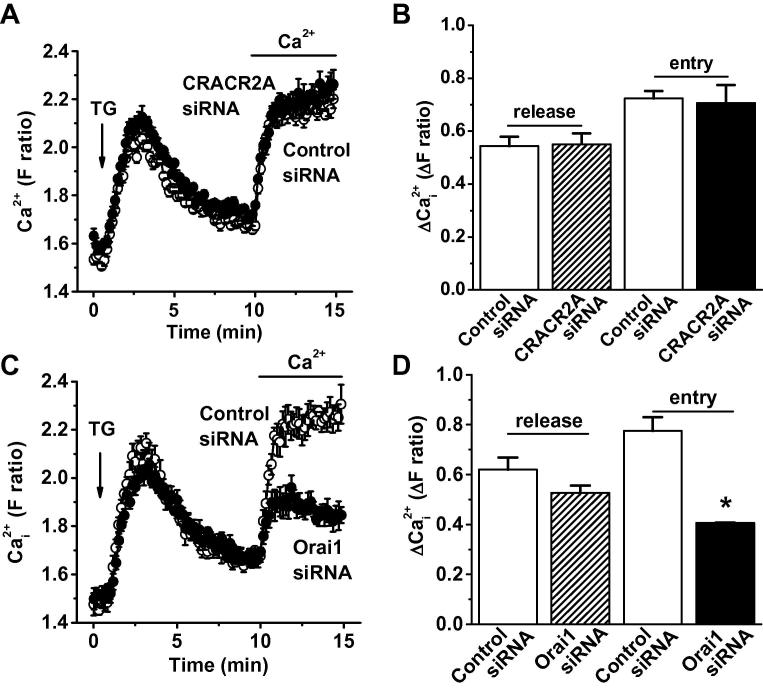
No effect of CRACR2A siRNA on CRAC channels in endothelial cells. (A) Mean ± s.e.m. paired intracellular Ca^2+^ data for human umbilical vein endothelial cells (HUVECs) transfected with control siRNA or CRACR2A siRNA (*N* = 4 each). Thapsigargin (TG, 1 μM) was added in the absence of extracellular Ca^2+^ before 2 mM Ca^2+^ was returned to the medium. (B) Summary data for 5 independent experiments of the type illustrated in (A). (C, D) As for (A, B) except using Orai1 siRNA in place of CRACR2A siRNA (*n*/*N* = 2/9).

Intracellular Ca^2+^ was recorded from human umbilical vein endothelial cells (HUVECs) to observe Ca^2+^ release evoked by thapsigargin (TG) in the absence of extracellular Ca^2+^ and then CRAC channel-mediated Ca^2+^ entry as extracellular Ca^2+^ was added back (Fig. 1A). Unexpectedly, transfection with short interfering RNA (siRNA) targeted to CRACR2A failed to affect Ca^2+^ release or Ca^2+^ entry (Fig. 1A and B). In contrast, siRNA targeted to Orai1 suppressed Ca^2+^ entry but not Ca^2+^ release, consistent with Orai1-dependent CRAC channels mediating Ca^2+^ entry (Fig. 1C and D). The data suggest that CRACR2A is unimportant for CRAC channels in these cells.

**Fig. 2:**
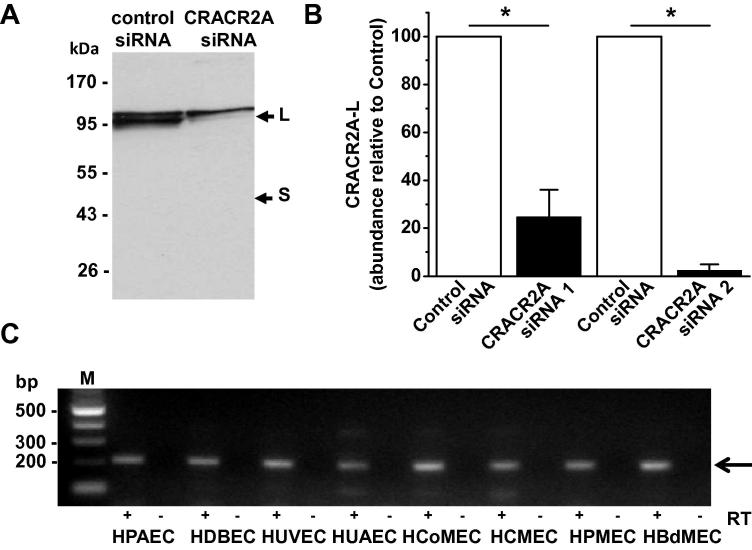
Detection of long but not short CRACR2A in endothelial cells. (A) Western blot probed with anti-CRACR2A antibody for HUVECs transfected with control or CRACR2A siRNA. S indicates the expected mass for CRACR2A (short variant). L indicates a larger protein (long variant) which was depleted by CRACR2A siRNA. Equal total protein was loaded in each lane. (B) Densitometry analysis for the large protein (L) seen in blots of the type shown in (A). Control siRNA was compared in paired experiments with CRACR2A siRNA 1 or CRACR2A siRNA 2 (*n* = 3 each). The band intensity in the CRACRA siRNA group is normalized to that in its control siRNA group. Equal total protein loading in each lane was validated by using anti-β-actin antibody. (C) PCR products with (+) or without (−) reverse transcriptase (RT) and using primers to the extended 3′ sequence of CRACR2A-L. Reactions are shown for mRNA isolated from human endothelial cells from pulmonary artery (HPAEC), dermal blood (HDBEC), umbilical artery (HUAEC), colon microv asculature (HCoMEC), cardiac microvasculature (HCMEC), pulmonary microvasculature (HPMEC), dermal microvasculature (DMEC) or bladder microvasculature (HBdMEC). The expected product was 214 bp.

An explanation for the above data could be that CRACR2A is not expressed. Indeed, anti-CRACR2A antibody failed to detect protein of ∼45 kDa (Fig. 2A), which is the expected mass of CRACR2A [3]. However, a doublet around 95 kDa was labelled by the antibody and the lower of these two bands was depleted by two different siRNAs targeted to CRACR2A (Fig. 2A and B). The upper band may reflect an unrelated protein, labelled non-specifically by the antibody. The data suggest that CRACR2A is expressed in endothelial cells but that it occurs at about twice the expected molecular mass.

To obtain an explanation for the larger mass we searched genome sequence databases and found that the gene encoding CRACR2A, *EFCAB4B*, is predicted to be alternatively spliced to give two variants. Alignment of the predicted amino acid sequences of the short (CRACR2A) and long (CRACR2A-L) variants revealed that the N-terminal components are identical but that CRACR2A-L contains a distinct long C-terminus bearing little or no similarity to CRACR2A (Fig. S1). The analysis suggests that the ∼95 kDa protein might be CRACR2A-L.

To determine if endothelial cells express transcript encoding CRACR2A-L we performed full-length cloning based on the predicted sequence and using HUVEC mRNA. Sequencing showed the clone to be identical to the sequence predicted to encode CRACR2A-L except for one codon for an additional amino acid between serine 424 and glutamine 425 (Fig. S1). The extra codon was also detected in human dermal microvascular endothelial cells and RT-PCR detected mRNA encoding CRACR2A-L in endothelial cells from 6 additional sources (Fig. 2C). The data suggest that the long variant, CRACR2A-L, is the variant of endothelial cells.

**Fig. 3:**
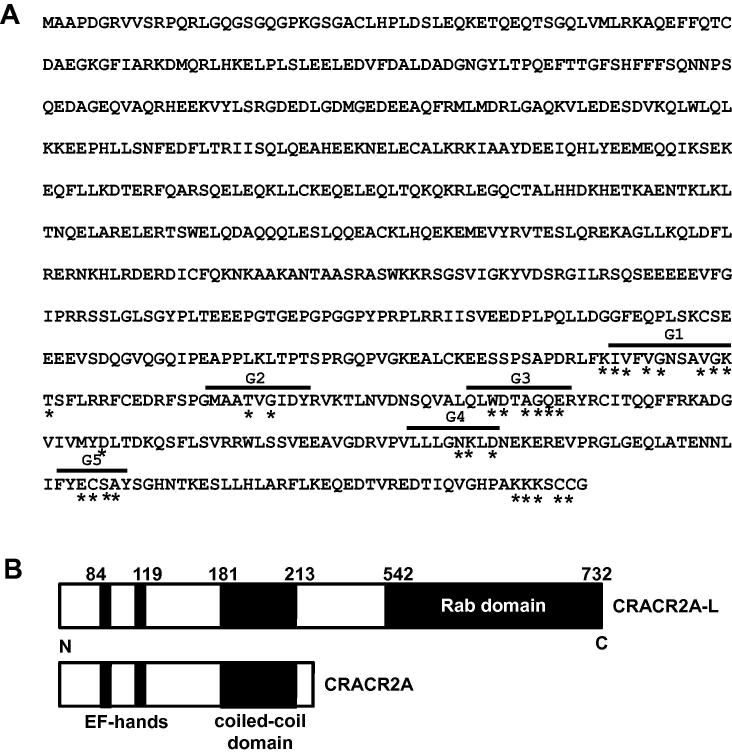
Sequence and Rab GTPase domains of CRACR2A-L. (A) Amino acid sequence for CRAC2RA-L. Over-lined are G boxes containing consensus residues (*) which are conserved in Rab proteins. (B) Diagram indicating features of CRACR2A-L (upper) and CRACR2A (lower). The N-terminus (N) and C-terminus (C) are indicated. The numbers are the amino acid positions in the total sequence.

Analysis of the distinct long C-terminus of CRACR2A-L indicated G boxes and other sequence similarities characteristic of Rab GTPases (Fig. 3). Construction of a dendrogram placed CRACR2A-L as a previously unrecognised member of the large Rab protein family (Fig. S2). It is relatively unusual amongst the Rab proteins in containing EF hands (Fig. 3), Ca^2+^-binding motifs that have been shown to be functional in CRACR2A [3].

**Fig. 4:**
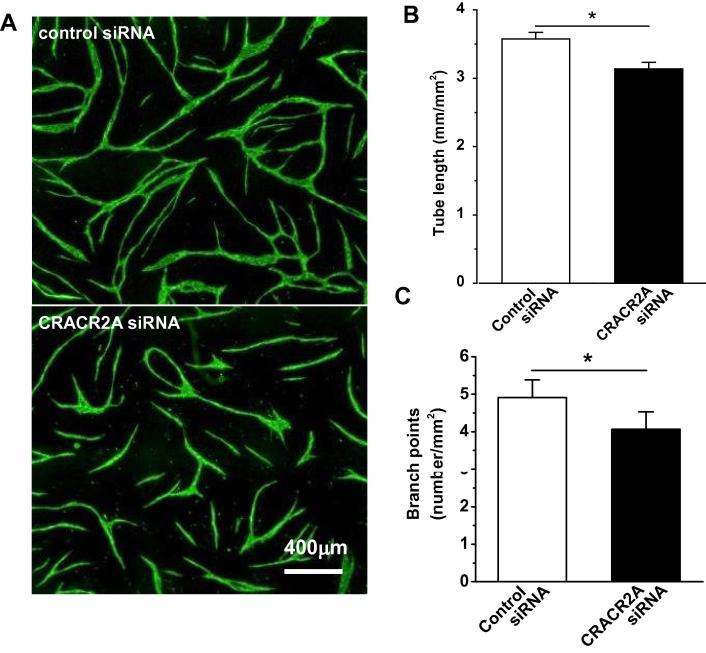
CRACR2A-L depletion suppresses endothelial tube formation. (A) Images of HUVECs labelled with anti-CD31 antibody (green) after forming tube-like structures on fibroblasts, which were not labelled. HUVECs were transfected with control siRNA or CRACR2A siRNA. (B) Summary histograms showing the effects of CRACR2A siRNA on tube length and number of branch points (*n*/*N* = 3/21). (For interpretation of the references to colour in this figure legend, the reader is referred to the web version of this article.)

To determine if CRACR2A-L has functional significance in endothelial cells we performed an assay in which HUVECs form tube, capillary-like, structures on a bed of fibroblasts, mimicking aspects of angiogenesis (Fig. 4A). Depletion of CRACR2A-L did not prevent tube formation but reduced the overall length of tubes and the number of branch points (Fig. 4B and C). The data suggest that CRACR2A-L is a functional protein of endothelial cells.

## 4 Discussion

The results suggest a previously unrecognized protein of the Rab GTPase family. It is encoded by the same gene as CRACR2A, a protein expressed in T cells and regulating CRAC channels, but alternative splicing enables a long C-terminal extension and total protein of about twice the size of CRACR2A. We refer to it as CRACR2A-L. The N-terminus is identical to CRACR2A. It is the additional C-terminal sequence that makes it a member of the Rab GTPase family. CRACR2A-L is expressed in endothelial cells, whereas CRACR2A is not. CRACR2A-L has no effect on CRAC channels in these cells and so we assume that the C-terminal sequence directs the protein away from these channels.

Rab proteins comprise the largest sub-group of the RAS superfamily and are best known for roles in intracellular trafficking [5,6]. Most Rab proteins are 20–25 kDa [7] and thus smaller than CRACR2A-L. Rab44 and Rab45 are, by contrast, similar in size to CRACR2A-L at 108 and 83 kDa [5,8]. As in CRACR2A-L the extra length appears to exist for incorporation of EF-hands and thus Ca^2+^ regulation. We speculate therefore that CRACR2A-L might be a mechanism for coupling cytosolic Ca^2+^ elevation to protein trafficking which in turn impacts on endothelial tube formation and potentially other as yet unidentified downstream processes. It would be challenging to test this idea through studies of tube formation because the impact of CRACR2A-L on this process was relatively small. Therefore, it will be important to identify specific downstream effectors and other downstream implications of CRACR2A-L in order to understand its suggested integrative role.

It is intriguing why the gene encoding this CRACR2A-L protein would also be capable of generating the shorter CRACR2A protein which regulates the Ca^2+^-permeable CRAC channels [3]. Although there was only CRACR2A-L in endothelial cells, regulation of alternative splicing may lead to co-expression of CRACR2A and CRACR2A-L in some contexts. In such contexts, CRACR2A would be expected to have a positive effect on CRAC channels, elevating the cytosolic Ca^2+^ concentration and potentially modulating the function of CRACR2A-L, integrating Ca^2+^-signalling and protein trafficking via a single gene.

We observed mild but significant positive impact of CRACR2A-L on endothelial cell remodelling. Additional functions of CRACR2A-L can be expected to emerge, however, and it would be premature to suggest that regulation of endothelial cell remodelling is its primary function.
