# Supplementary material for: Expression of a long variant of CRACR2A that belongs to the Rab GTPase protein family in endothelial cells
Source: Biochem Biophys Res Commun. 2015 Jan 2;456(1):398–402. doi: 10.1016/j.bbrc.2014.11.095 (PMC4300414; doi:10.1016/j.bbrc.2014.11.095)
Supplement: Supplementary data 1 [file mmc1.pdf]

SUPPLEMENTARY INFORMATION

Expression of a long variant of CRACR2A that belongs to the Rab GTPase protein family in endothelial cells

Lesley A Wilson+, Lynn McKeown+, Sarka Tumova, Jing Li, David J Beech*
School of Medicine, University of Leeds, Leeds, LS2 9JT, UK.

+Equal contributors

*Author for correspondence: Prof David J Beech, School of Medicine, Garstang Building, Mount Preston Street, University of Leeds, Leeds, LS2 9JT, England, UK; d.j.beech@leeds.ac.uk; Tel: +44-(0)-113-343-4323.

Figure S1. Amino acid sequence alignment for CRACR2A and CRACR2A-L. Identity is indicated by *. The arrow points to the additional serine residue in the sequence cloned from HUVECs.

Figure S2. Dendrogram for Rab proteins and including CRAC2RA-L. Rab protein sequences were aligned using Clustal Omega software. The generated Newick tree file was up loaded to Phy.fi [1] to produce the phylogenic tree. The position of CRACR2A-L is highlighted by an arrow.

Reference

[1] J. Fredslund, PHY.FI: fast and easy online creation and manipulation of phylogeny color figures, BMC Bioinformatics 7 (2006) 315.

```
CRACR2A         MAAPDGRVVSRPQRLGQGSGQGPKGSGACLHPLDSLEQKETQEQTSGQLVMLRKAQEFFQ 60
CRACR2A-L       MAAPDGRVVSRPQRLGQGSGQGPKGSGACLHPLDSLEQKETQEQTSGQLVMLRKAQEFFQ 60
                ************************************************************

CRACR2A         TCDAEGKGFIARKDMQRLHKELPLSLEELEDVFDALDADGNGYLTPQEFTTGFSHFFFSQ 120
CRACR2A-L       TCDAEGKGFIARKDMQRLHKELPLSLEELEDVFDALDADGNGYLTPQEFTTGFSHFFFSQ 120
                ************************************************************

CRACR2A         NNPSQEDAGEQVAQRHEEKVYLSRGDEDLGDMGEDEEAQFRMLMDRLGAQKVLEDESDVK 180
CRACR2A-L       NNPSQEDAGEQVAQRHEEKVYLSRGDEDLGDMGEDEEAQFRMLMDRLGAQKVLEDESDVK 180
                ************************************************************

CRACR2A         QLWLQLKKEEPHLLSNFEDFLTRIISQLQEAHEEKNELECALKRKIAAYDEEIQHLYEEM 240
CRACR2A-L       QLWLQLKKEEPHLLSNFEDFLTRIISQLQEAHEEKNELECALKRKIAAYDEEIQHLYEEM 240
                ************************************************************

CRACR2A         EQQIKSEKEQFLLKDTERFQARSQELEQKLLCKEQELEQLTQKQKRLEGQCTALHHDKHE 300
CRACR2A-L       EQQIKSEKEQFLLKDTERFQARSQELEQKLLCKEQELEQLTQKQKRLEGQCTALHHDKHE 300
                ************************************************************

CRACR2A         TKAENTKLKLTNQELARELERTSWELQDAQQQLESLQQEACKLHQEKEMEVYRVTESLQR 360
CRACR2A-L       TKAENTKLKLTNQELARELERTSWELQDAQQQLESLQQEACKLHQEKEMEVYRVTESLQR 360
                ************************************************************

CRACR2A         EKAGLLKQLDFLR----------------------------------------------- 373
CRACR2A-L       EKAGLLKQLDFLRERNKHLRDERDICFQKNKAAKANTAASRASWKKRSGSVIGKYVDSRG 420
                *************

CRACR2A         ---------------------------------------CVGGHWP-------------- 380
CRACR2A-L       ILRSSQSEEEEEVFGIPRRSSLGLSGYPLTEEEPGTGEPGPGGPYPRPLRRIISVEEDPL 480
                                                         ** :*

CRACR2A         -----------------------------VLRAPPRSLGSEGPV---------------- 395
CRACR2A-L       PQLLDGGFEQPLSKCSEEEEVSDQGVQGQIPEAPPLKLTPTSPRGQPVGKEALCKEESSP 540
                                             : .*** .* . .*

CRACR2A         ------------------------------------------------------------
CRACR2A-L       SAPDRLFKIVFVGNSAVGKTSFLRRFCEDRFSPGMAATVGIDYRVKTLNVDNSQVALQLW 600

CRACR2A         ------------------------------------------------------------
CRACR2A-L       DTAGQERYRCITQQFFRKADGVIVMYDLTDKQSFLSVRRWLSSVEEAVGDRVPVLLLGNK 660

CRACR2A         ------------------------------------------------------------
CRACR2A-L       LDNEKEREVPRGLGEQLATENNLIFYECSAYSGHNTKESLLHLARFLKEQEDTVREDTIQ 720

CRACR2A         ------------
CRACR2A-L       VGHPAKKKSCCG 732
```

Figure S1

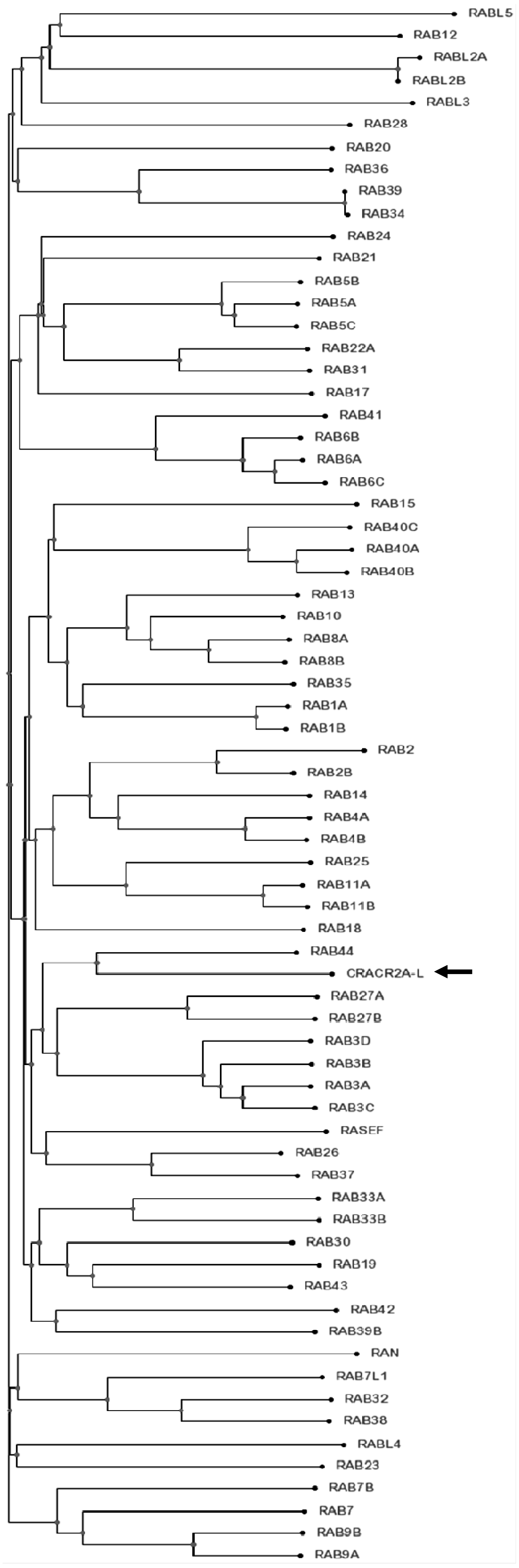
RABL5
RAB12
RABL2A
RABL2B
RABL3
RAB28
RAB20
RAB36
RAB39
RAB34
RAB24
RAB21
RAB5B
RAB5A
RAB5C
RAB22A
RAB31
RAB17
RAB41
RAB6B
RAB6A
RAB6C
RAB15
RAB40C
RAB40A
RAB40B
RAB13
RAB10
RAB8A
RAB8B
RAB35
RAB1A
RAB1B
RAB2
RAB2B
RAB14
RAB4A
RAB4B
RAB25
RAB11A
RAB11B
RAB18
RAB44
CRACR2A-L
RAB27A
RAB27B
RAB3D
RAB3B
RAB3A
RAB3C
RASEF
RAB26
RAB37
RAB33A
RAB33B
RAB30
RAB19
RAB43
RAB42
RAB39B
RAN
RAB7L1
RAB32
RAB38
RABL4
RAB23
RAB7B
RAB7
RAB9B
RAB9A

Figure S2
